# Gender Differences in Burnout Among Endocrinologists in China

**DOI:** 10.3389/fpsyg.2022.845188

**Published:** 2022-03-01

**Authors:** Jing Wang, Lufa Zhang, Feng Jiang, Yuanli Liu, Mingxiao Wang, Yinuo Wu, Yi-Lang Tang

**Affiliations:** ^1^China Institute for Urban Governance, Shanghai Jiao Tong University, Shanghai, China; ^2^School of International and Public Affairs, Shanghai Jiao Tong University, Shanghai, China; ^3^Institute of Healthy Yangtze River Delta, Shanghai Jiao Tong University, Shanghai, China; ^4^School of Health Policy and Management, Chinese Academy of Medical Sciences & Peking Union Medical College, Beijing, China; ^5^Department of Cardiology, Emergency General Hospital, Beijing, China; ^6^Chinese Academy of Medical Sciences & Peking Union Medical College, Beijing, China; ^7^Department of Psychiatry and Behavioral Sciences, Emory University, Atlanta, GA, United States; ^8^Atlanta VA Medical Center, Decatur, GA, United States

**Keywords:** gender differences, burnout, endocrinologists, associated factors, prevalence

## Abstract

**Objective:**

To survey the prevalence of burnout in a national sample of endocrinologists in China and to examine its correlates, with a special focus on gender differences.

**Methods:**

An anonymous online survey was conducted among endocrinologists in 31 provincial government-owned “People’s Hospitals” of each province in mainland China. Demographic and work-related factors were collected from participants. The Maslach Burnout Inventory-Human Services Survey (MBI-HSS) was used to assess burnout, including emotional exhaustion (EE), depersonalization (DP), and reduced personal accomplishment (PA).

**Results:**

A total of 711 endocrinologists (72.1% were female and mean age was 39.63 ± 8.51 years old) completed the survey. Burnout was reported by 32.8% of the participants. There were no significant gender differences in the overall prevalence of burnout or EE, DP, and PA (all *p* > 0.05). A multi-level linear regression revealed: (1) In male participants, PA was significantly associated with age (β = 0.03, *p* = 0.003), DP was inversely associated with age (β = −0.06, *p* = 0.005), EE was significantly associated with shorter sleep duration (β = −0.25, *p* = 0.006), and longer work hours (β = 0.01, *p* = 0.016). (2) In females, PA was significantly associated with age (β = 0.01, *p* = 0.038), EE and DP were both significantly associated with shorter sleep duration (β = −0.19, *p* = 0.001; and β = −0.15, *p* = 0.011, respectively). EE and DP were also associated with work hours (β = 0.02, *p* < 0.001; and β = 0.01, *p* < 0.001, respectively).

**Conclusion:**

Nearly one-third of endocrinologists in China experienced burnout. Although there were no significant gender differences in the prevalence of overall burnout or EE, DP, and PA scores, male and female participants differed in factors associated with EE, DP, and PA. Interventions need to be tailored to target different aspects in male and female endocrinologists and target different subgroups.

## Introduction

The concept of burnout in healthcare professionals has been used to describe the emotional and psychological status related to job stress (Rotenstein et al., 2018). According to Maslach’s foundational work in the 1980s, burnout generally has been defined as a combination of emotional exhaustion (EE), depersonalization (DP), and reduced personal accomplishment (PA) (West et al., 2018). Factors that have been shown to contribute to burnout include excessive workloads, long working hours, frequent call duties; and individual factors, such as poor coping strategies, perfectionism, educational debt, relationship status, sleep deprivation, etc. (Shanafelt et al., 2015; Patel et al., 2018; West et al., 2018; Hacimusalar et al., 2021; Lin et al., 2021). A series of studies have found that burnout in physicians was associated with increased medical errors, poor patient satisfaction, and decreased physicians’ health and safety (Panagioti et al., 2018; Dyer, 2019; Hu et al., 2019; Wu et al., 2020). Therefore, it is essential to ascertain the prevalence and associated factors of burnout in physicians. However, the findings of existing studies have shown a wide range in burnout prevalence, likely due to assessment methods, different samples, and study quality (Rotenstein et al., 2018).

Endocrinologists have played a uniquely important role in the Chinese healthcare system, owing to a very high prevalence of diabetes and other related metabolic diseases (Li et al., 2020; Li Y. et al., 2021; Zhang L. et al., 2021). Like all healthcare professionals, endocrinologists are not immune to physician burnout and stress. According to the *Medscape Endocrinologist Lifestyle, Happiness & Burnout Report 2020*, 46% of endocrinologists in the United States reported burnout, which was higher than the overall level of all physicians (41%) (Medscape, 2020). A previous study demonstrated that the overall burnout rate among physicians from Chinese general hospitals was 31.28% (Wu et al., 2020), while the prevalence of burnout in Chinese endocrinologists is still unclear.

Gender difference in burnout has been an interest of research for the past two decades. Many early researchers argued that burnout is more of a female experience (Maslach et al., 2001). In healthcare settings, several studies showed significant gender differences in physician burnout prevalence and associated factors. A few studies found that female health workers had a higher risk of burning out (Aguwa et al., 2014; Granek et al., 2016; Eden et al., 2020; Loscalzo et al., 2021; McPeek-Hinz et al., 2021), and some suggested that educational level, socioeconomic status and working hours were associated with burnout in female physicians (Norlund et al., 2010). On the contrary, a few studies found no gender differences in physician burnout (Linzer et al., 2002; te Brake et al., 2003; Doraiswamy et al., 2021).

This study aimed to survey the prevalence of burnout in a national sample of endocrinologists in China and to examine its correlates, with a special focus on gender differences. We hypothesized that there would be gender differences in the rate of burnout, and in the association between burnout and correlates among Chinese endocrinologists.

## Materials and Methods

### Study Design and Participants

A national cross-sectional survey was conducted from March 18 to 31, 2019. The study was a part of the China Healthcare Improvement Initiative, which was supported by the National Health Committee (Zhou et al., 2018). We purposely invited the “People’s Hospital” of each province in mainland China, as they are the most prominent hospitals affiliated with each provincial government. All of them were general tertiary hospitals (National Health Commission of the People’s Republic of China, 2020). Thirty-one hospitals participated, and all endocrinologists who were working in those hospitals at that time were invited to participate in the anonymous online survey. To reach all endocrinologists, we collaborated with the hospital administrators of these hospitals to organize the endocrinologists to participate in this survey. The weblink of the survey was delivered through WeChat, a popular social app in mainland China.

The research protocol was approved by the Ethics Committee of the Emergency General Hospital in Beijing. Each participant completed the consent form before they began the questionnaire.

### Measures

Burnout was assessed using the 22-item Maslach Burnout Inventory-Human Services Survey (MBI-HSS)(Maslach and Jackson, 1986). This scale has been considered the gold standard tool for burnout measuring (Verweij et al., 2017). The Chinese version of MBI-HSS has been shown to have good reliability and validity (Li et al., 2003). The Cronbach’s α was 0.96 in this study.

It consists of three subscales: EE–nine items; DP- five items; and reduced PA- eight items. Each item was scored on a 7-point Likert scale, ranging from 0 (stands for never) to 6 (stands for always). We defined overall burnout as EE score ≥27 and/or DP score ≥10, aligned with one of the most popular criteria (Rotenstein et al., 2018). The Cronbach’s α was 0.91 for EE, 0.79 for DP, and 0.89 for PA, respectively.

Additionally, we collected the basic sociodemographic data (age, sex, marital status, education, number of children) and work-related factors, such as professional title (junior, middle, and senior), administration position (yes or no), working hours per week, night shifts per month, and sleep hours per day, based on literature review (te Brake et al., 2003; Norlund et al., 2010; Rotenstein et al., 2018).

### Data Analysis

One-sample K-S test was used to detect the normality of obtained data. Descriptive analyses were used to describe the sample’s socio-demographic, burnout symptoms, and work-related factors.

Since there is no consensus on the diagnostic criteria for burnout syndromes, some suggested the three subscales should be treated as continuous measures (Rotenstein et al., 2018). We adopted this approach in our analysis. The Chi-square test examined the gender difference of burnout prevalence. The Kruskal–Wallis test or Pearson correlation analysis was conducted to test the correlation between related factors and EE, DP, and PA in male and female participants. After that, significant factors were involved in further regression analysis. As all endocrinologists nested in hospitals, multilevel linear regression analyses were conducted to identify independent factors associated with EE, DP, PA in male and female samples, respectively.

We performed all statistical analyses using the STATA software version 16.0 (Stata Corporation, College Station, TX, United States), with the significance level at the *p*-value of 0.05 (two-tailed).

## Results

### Sample Characteristics

In total, all endocrinologists (*N* = 879) nested in these 31 hospitals were invited to respond to this survey, and 711 endocrinologists (response rate = 80.89%) completed the questionnaire. Table 1 shows the detailed information of their sociodemographic characteristics, job-related factors, and gender differences.

**TABLE 1:** Basic Characteristics of 711 endocrinologists in China.

Characteristic		N (%)	Male (198)	Female (513)	*p*
Relationship status					0.809
	Not married	75 (10.55)	20 (10.10)	55 (10.72)	
	Married	636 (89.45)	178 (89.90)	458 (89.28)	
Children					**0.008**
	None	149 (20.96)	32 (16.16)	117 (22.81)	
	One	457 (64.28)	125 (63.13)	332 (64.72)	
	More than one	105 (14.77)	41 (20.71)	64 (12.48)	
Educational level*					0.222
	Medical/college degree	122 (17.16)	36 (18.18)	86 (16.76)	
	Add on Master’s degree	356 (50.07)	89 (44.95)	267 (52.05)	
	Add on Doctorate degree	233 (32.77)	73 (36.87)	160 (31.19)	
Professional title					**0.001**
	Junior	119 (16.74)	21 (10.61)	98 (19.10)	
	Middle	252 (35.44)	61 (30.81)	191 (37.23)	
	Senior	340 (48.82)	116 (58.59)	224 (43.66)	
Administration position					**<0.001**
	No	606 (85.23)	151 (76.26)	455 (88.69)	
	Yes	105 (14.77)	47 (23.74)	58 (11.31)	
		Mean (SD)	Mean (SD)	Mean (SD)	*p*
Age (years)		39.63 (8.51)	42.55 (8.49)	38.50 (8.26)	**<0.001**
Sleep hours/day		6.35 (0.76)	6.41 (0.73)	6.33 (0.78)	0.261
Work hours/week		55.06 (13.83)	54.81 (13.18)	55.16 (14.08)	0.762
		Median (IQR)	Median (IQR)	Median (IQR)	*p*
Night shifts/month		6 (1)	4 (3)	4 (2.25)	0.137

*In China, medical school graduates are awarded a bachelor’s degree in medicine (similar to the European system). Some obtain a master’s or doctorate degree in addition to their medical degree. Bold value for p < 0.05.

According to the Labor Law of China, workers should work no more than 44 h per week (Standing Committee of National People’s Congress, 2019). However, nearly four-fifths (79.3%) of the endocrinologists in our sample reported working more than 44 h per week, with no significant gender difference (79.8 in males vs. 79.1% in females, *p* = 0.847).

The overall prevalence of burnout was 32.7% in this sample, again with no significant gender difference (34.3% in males and 32.2% in females, *p* = 0.579). There were no gender differences in EE, DP, PA scores either (all *p* > 0.05) (Table 2).

**TABLE 2:** Burnout in male (*N* = 198) and female (*N* = 513) endocrinologists in China.

	Total	Male	Female	*p*
	**Mean (Median, IQR)**	**Mean (Median, IQR)**	**Mean (Median, IQR)**	

EE	16.71 (15,15)	15.91 (14.5,14)	17.02 (15,14)	0.249^a^
DP	7.29 (6,7)	7.24 (6,7)	7.30 (6,7)	0.875^a^
PA	32.24 (33,17)	31.80 (33,19)	32.41 (34,16)	0.670^a^
	N (%)	N (%)	N (%)	
Burnout	233 (32.77%)	68 (34.34%)	165 (32.16%)	0.579^b^

*^a^Kruskal-Wallis test.*

*^b^Chi square test.*

Univariate analyses showed that professional title was associated with EE, DP in males, and DP in females; administration position was only associated with PA in females. Age, sleep duration, work hours, and the number of night shifts was associated with some of the EE, DP, and DP scores among endocrinologists (Table 3).

**TABLE 3:** Univariate analyses of factors associated with burnout in 711 endocrinologists in China.

Variable		Male						Female					
		EE		DP		PA		EE		DP		PA	
		Median (IQR)	*p*	Median (IQR)	*p*	Median (IQR)	*p*	Median (IQR)	*p*	Median (IQR)	*p*	Median (IQR)	*p*
Relationship status			0.209		0.171		0.915		0.848		0.748		0.883
	Not married	17.5 (15)		7.5 (7.5)		34 (8.5)		14 (14)		6 (7)		33 (13)	
	Married	14 (14)		6 (7)		32 (19)		15 (14)		6 (7)		34 (16)	
Children			0.251		0.352		0.404		0.643		0.255		0.457
	None	19 (16)		7 (7.5)		32.5 (17)		15 (15)		6 (6)		35 (14)	
	One	13 (14)		6 (7)		34 (18)		15 (15)		6 (7)		32.5 (17)	
	More than one	15 (12)		5 (7)		30 (22)		14 (12)		5 (7.5)		36 (17.5)	
Educational level			0.396		0.231		0.715		0.086		0.558		0.524
	Medical/college degree	12 (12)		4 (7)		31.5 (18)		13 (13)		6 (6)		34 (19)	
	Add on Master’s degree	15 (16)		6 (7)		33 (16)		16 (16)		6 (7)		32 (16)	
	Add on Doctorate degree	14 (14)		6 (8)		34 (18)		13 (14)		6 (7)		36 (17)	
Professional title			**0.007**		**0.007**		0.103		0.256		**0.027**		0.657
	Junior	22 (8)		10 (7)		29 (14)		14 (15)		6 (5)		34 (16)	
	Middle	16 (19)		5 (7)		33 (18)		16 (20)		7 (9)		32 (16)	
	Senior	12 (14)		6 (7)		33 (18.5)		14 (11.5)		5 (6)		35 (17.5)	
Administration position			0.125		0.489		0.908		0.143		0.248		**0.020**
	No	15 (16)		6 (7)		33 (18)		15 (15)		6 (7)		33 (16)	
	Yes	12 (14)		6 (7)		31 (20)		13 (13)		5 (5)		39.5 (17)	
		*r*	*p*	*r*	*p*	*r*	*p*	*r*	*p*	*r*	*p*	*r*	*p*
Age (years)		−0.27	**<0.001**	−0.27	**<0.001**	0.21	**0.003**	−0.06	0.203	−0.11	**0.014**	0.12	**<0.001**
Sleep hours/day		−0.18	**0.009**	−0.04	0.556	−0.07	0.341	−0.20	**<0.001**	−0.13	**0.003**	0.06	0.161
Work hours/week		0.26	**<0.001**	0.15	**0.030**	−0.07	0.300	0.31	**<0.001**	0.25	**<0.001**	−0.03	0.494
Night shifts/month		0.16	**0.024**	0.13	0.061	−0.14	0.054	0.15	**<0.001**	0.09	**0.049**	−0.05	0.219

*Bold value for p < 0.05.*

Furthermore, EE, DP, and DP scores were transformed into the standard normal distribution (Z scores). In multilevel linear regression analysis for male and female endocrinologists, standardized EE, DP, and DP scores were used as dependent variables, while the significant variables identified in univariate analyses were enrolled as independent variables. Table 4 demonstrates that shorter sleep duration was significantly associated with EE in males (β = −0.25, *p* = 0.006), while work hours were significantly associated with male EE (β = 0.01, *p* = 0.016). Additionally, age was significantly associated with male PA (β = 0.03, *p* = 0.003), but was inversely related to male DP (β = −0.06, *p* = 0.005).

**TABLE 4:** Multilevel analysis of associated factors for burnout among 198 male endocrinologists.

Variable		EE*				DP*				PA*			
		β	95% CI (Lower)	95% CI (Upper)	*p*	β	95% CI (Lower)	95% CI (Upper)	*p*	β	95% CI (Lower)	95% CI (Upper)	*p*
Age (years)		−0.02	−0.05	0.00	0.059	−0.03	−0.06	−0.01	**0.005**	0.03	0.01	0.04	**0.003**
Sleep hours/day		−0.25	−0.42	−0.07	**0.006**	–	**–**	**–**	**–**	–	–	–	–
Work hours/week		0.01	0.00	0.02	**0.016**	0.01	0.00	0.02	0.256	–	–	–	–
Night shifts/month		0.01	−0.05	0.06	0.765	–	**–**	**–**	**–**	–	–	–	–
Professional title (ref. Junior)													
	Middle	−0.18	−0.65	0.30	0.465	−0.37	−0.86	0.11	0.130	–	–	–	–
	Senior	−0.12	−0.67	0.44	0.680	−0.13	−0.70	0.44	0.650	–	–	–	–

*Transformed into standardized normal variate. Bold value for p < 0.05.

Table 5 shows that shorter sleep duration was associated with female EE (β = −0.19, *p* = 0.001) and DP (β = −0.15, *p* = 0.011). Work hours were significantly associated with EE (β = 0.02, *p* < 0.001) and DP (β = 0.01, *p* < 0.001) in females. Age was also significantly associated with PA in females (β = 0.01, *p* = 0.038).

**TABLE 5:** Multilevel analysis of associated factors for burnout among 513 female endocrinologists.

Variable		EE*				DP*				PA*			
		β	95% CI (Lower)	95% CI (Upper)	*p*	β	95% CI (Lower)	95% CI (Upper)	*p*	β	95% CI (Lower)	95% CI (Upper)	*p*
Age (years)		–	–	–	–	−0.01	−0.03	0.01	0.193	0.01	0.00	0.02	**0.038**
Sleep hours/day		−0.19	−0.29	−0.08	**0.001**	−0.15	−0.26	−0.03	**0.011**	–	–	–	–
Work hours/week		0.02	0.01	0.03	**<0.001**	0.01	0.01	0.02	**<0.001**	–	–	–	–
Night shifts/month		0.02	−0.01	0.05	0.177	−0.01	−0.04	0.03	0.723	–	–	–	–
Professional title (ref. Junior)													
	Middle	–	–	–	–	0.24	0.00	0.49	0.054	–	–	–	–
	Senior	–	–	–	–	0.10	−0.25	0.45	0.563	–	–	–	–
Administration position (ref. No)		–	–	–	–	–	–	–	–	0.17	−0.11	0.45	0.229

*Transformed into standardized normal variate. Bold value for p < 0.05.

## Discussion

This study was one of the first to survey the prevalence of burnout and the associated factors among Chinese endocrinologists. Our main findings include: (1) nearly one-third of participants reported burnout, and there was no significant gender difference in the overall prevalence of burnout; (2) a younger age was associated with DP in males, but not in females; and (3) DP was significantly associated with shorter sleep duration and longer work hours in females, but not in males. This suggested that there might be gender differences in the mechanism of burnout between burnout (DP) and related factors.

In this study, we found that age was an independent factor associated with PA, while sleep hours and work hours were significantly associated with EE among male and female endocrinologists, which is in concordance with previous studies (West et al., 2018; Hacimusalar et al., 2021; Lin et al., 2021).

Our finding that there were no significant gender differences in the overall prevalence of burnout is different from most previous studies. Numerous studies showed that female physicians had a higher prevalence of burnout from countries including America, China, Sweden, Nigeria, etc. (Norlund et al., 2010; Dyrbye et al., 2011; Aguwa et al., 2014; Granek et al., 2016; Huang et al., 2019; Eden et al., 2020; Gold et al., 2021; Lee et al., 2021; McPeek-Hinz et al., 2021). A few other studies reported only marginal gender differences in physician burnout prevalence (Linzer et al., 2002; te Brake et al., 2003; Śliwiński et al., 2014; El Ghaziri et al., 2019; Doraiswamy et al., 2021). The differences may be due to sampling, research settings, cultures, assessment methods for burnout, and variable cut-off values—at least 142 unique burnout definitions or subscale criteria (Rotenstein et al., 2018). For example, previous studies have used the Shirom Melamed Burnout Questionnaire (Norlund et al., 2010), Freudenberger Burnout Scale (Aguwa et al., 2014), Mini-Z burnout scale (Gold et al., 2021), Utrechtse Burnout Schaal (te Brake et al., 2003), or Burnout Scale Inventory (Śliwiński et al., 2014). Others used one or two items to measure burnout (Granek et al., 2016; El Ghaziri et al., 2019; Eden et al., 2020; McPeek-Hinz et al., 2021). Huang et al. used a 7-point Likert scale for MBI-HSS, ranging from 1 to 7, instead of 0 to 6 (Ma et al., 2019). Meanwhile, Lee et al. (2021) used a 5-point Likert scale for MBI. The methodological heterogeneity may contribute to the variations among the studies. Our findings are consistent with a meta-analysis, which included 183 studies of different populations. They showed that the commonly held belief that female employees are more likely to experience burnout is not supported by data (Purvanova and Muros, 2010).

Additionally, previous studies demonstrated that the gender differences might be attributed to job-related and situational life factors (te Brake et al., 2003; Norlund et al., 2010). Our study showed no significant gender differences in most job-related factors between male and female endocrinologists. This may help explain why the overall prevalence and subscales of burnout male and female endocrinologists were comparable.

Depersonalization, which is defined as “a negative attitude toward customers and clients, a personal detachment, or loss of ideals,” is generally believed to result from low job satisfaction or poor work-life balance (Maslach, 1993). Some researchers suggested resilience and coping strategies play an important role in affecting DP (Chong et al., 2021; Di Giuseppe et al., 2021; Di Trani et al., 2021; Li P. et al., 2021; Nituica et al., 2021; Perry et al., 2021). Studies also showed gender differences in coping strategies and resilience (Gargiulo et al., 2021; Reisch et al., 2021; Yan et al., 2021; Zhang X. et al., 2021), and this could partially explain the gender differences regarding the differential associations between work hours and sleep duration and DP (Di Giuseppe et al., 2021). As the etiology and mechanism of burnout remain elusive, these findings would help understand the pathogenesis of physician burnout. In the meantime, hospital management and healthcare policymakers need to pay more attention to short sleep duration and long work hours among female physicians to reduce DP or burnout.

The present study has a few limitations. First, the findings were based on a cross-sectional survey, and the nature of the design makes it difficult to infer causality for most factors. Second, the study data obtained may have recall bias, as is inherent in self-report questionnaire studies. Third, as the sample was only from tertiary public hospitals in China, the generalizability of the study conclusions may be limited. Finally, some important information related to burnout, such as personality and resilience, were not collected.

## Conclusion

In a large national sample, we found that nearly one-third of endocrinologists experienced burnout and the subscales in the sample. Although we did not find gender differences in the overall prevalence of burnout in the sample, we found in male and female participants that the associated factors were different, which indicated the mechanism of burnout in males and females might be different. This suggests that when developing interventions, hospital management should consider gender differences, and pay close attention to various aspects and target different subgroups, such as ensuring enough sleep duration and shortening work hours for female endocrinologists.

## Data Availability Statement

The original contributions presented in the study are included in the article/supplementary material, further inquiries can be directed to the corresponding authors.

## Ethics Statement

The studies involving human participants were reviewed and approved by the Ethics Committee of the Emergency General Hospital in Beijing. The patients/participants provided their written informed consent to participate in this study.

## Author Contributions

YL and YW made substantial contributions to the study design. MW and YW collected data. JW and FJ analyzed the data. JW, LZ, and FJ interpreted the analysis results and completed the manuscripts. Y-LT contributed to data interpretation, presentation, and critical revision of the manuscript. All authors have read and approved the published version of the manuscript.

## Conflict of Interest

The authors declare that the research was conducted in the absence of any commercial or financial relationships that could be construed as a potential conflict of interest.

## Publisher’s Note

All claims expressed in this article are solely those of the authors and do not necessarily represent those of their affiliated organizations, or those of the publisher, the editors and the reviewers. Any product that may be evaluated in this article, or claim that may be made by its manufacturer, is not guaranteed or endorsed by the publisher.
